# Vitamin D increases programmed death receptor-1 expression in Crohn’s disease

**DOI:** 10.18632/oncotarget.15489

**Published:** 2017-02-18

**Authors:** Mia Bendix, Stinne Greisen, Anders Dige, Christian L. Hvas, Nina Bak, Søren P. Jørgensen, Jens F. Dahlerup, Bent Deleuran, Jørgen Agnholt

**Affiliations:** ^1^ Department of Hepatology and Gastroenterology, Aarhus University Hospital, Aarhus, Denmark; ^2^ Department of Immunology, Institute of Biomedicine, Aarhus University, Aarhus, Denmark; ^3^ Department of Rheumatology, Aarhus University Hospital, Aarhus, Denmark

**Keywords:** PD-1, Crohns disease, vitamin D, T cells, Immunology and Microbiology Section, Immune response, Immunity

## Abstract

Background: Vitamin D modulates inflammation in Crohns disease (CD). Programmed death (PD)-1 receptor contributes to the maintenance of immune tolerance. Vitamin D might modulate PD-1 signalling in CD.

Aim: To investigate PD-1 expression on T cell subsets in CD patients treated with vitamin D or placebo.

Methods: We included 40 CD patients who received 1200 IU vitamin D3 for 26 weeks or placebo and eight healthy controls. Peripheral blood mononuclear cells (PBMCs) and plasma were isolated at baseline and week 26. The expressions of PD-1, PD-L1, and surface activation markers were analysed by flow cytometry. Soluble PD-1 plasma levels were measured by ELISA.

Results: PD-1 expression upon T cell stimulation was increased in CD4^+^CD25^+int^ T cells in vitamin D treated CD patients from 19% (range 10 39%) to 29% (11 79%)(*p* = 0.03) compared with placebo-treated patients. Vitamin D treatment, but not placebo, decreased the expression of the T cell activation marker CD69 from 42% (31 62%) to 33% (19 - 54%)(*p* = 0.01). Soluble PD-1 levels were not influenced by vitamin D treatment.

Conclusions: Vitamin D treatment increases CD4^+^CD25^+int^ T cells ability to up-regulate PD-1 in response to activation and reduces the CD69 expression in CD patients.

## INTRODUCTION

Vitamin D has important immunomodulating functions, which may influence the natural course of autoimmune diseases. In Crohn's disease (CD), vitamin D deficiency is common [1, 2] and associated with both a low quality of life [3] and an increased disease activity [4, 5]. A randomised clinical trial indicated that oral vitamin D treatment reduces the relapse rate and disease activity [6]. Vitamin D's effect on CD may be due to the modulation of cellular immune responses. Following 26 weeks of oral vitamin D treatment to CD patients, T cells decreases the ability to up-regulate the vitamin D receptor (VDR) [7]. Further, *in vitro* 25-hydroxyvitamin D3 (25-vitD) and 1.25-dihydroxyvitamin D3 (1.25(OH)_2_D) stimulation reduces the differentiation of T-helper 17 (Th17) cells and decreases the production of interferon (IFN)-γ and interleukin (IL)-17 [8]. Also, *in vitro* 1α,25-dihydroxyvitamin D3 stimulation promotes regulatory T cells (Treg) function [9]. Still, the influence of oral vitamin D on T cells is not elucidated.

CD is characterised by transmural mucosal inflammation dominated by a loss of tolerance towards the commensal intestinal microbiota [10]. The regulation of immunological tolerance plays a critical role in CD [11]. Programmed death (PD)-1 is a co-inhibitory receptor, which is expressed by activated T cells, B cells, and Natural Killer cells [12] and is involved in maintenance of immune tolerance. Upon binding with the ligands PD-L1 and PD-L2, which are expressed by haematopoietic lineage cells and antigen presenting cells [13], PD-1 inhibits T cell function, survival, and activation [14]. By this way, PD-1 serves to hinder auto-reactive T cell responses [15]. PD-1 and the PD-1 ligands also exist in soluble forms. Soluble (s) PD-1 is elevated in several autoimmune diseases such as diabetes mellitus [16], systemic lupus erythematosus, and rheumatoid arthritis (RA) [17, 18]. In RA, sPD-1 levels are associated with increased disease activity and clinical scores [19].

PD-1 expression is increased in exhausted T cells, which are identified in patients with chronic infections and autoimmune diseases [20–22]. Exhausted T cells which contribute to peripheral tolerance [23] are associated with less flare-ups in IBD[21].

In mucosal lamina propria cells, PD-1 and PD-L1 expressions are up-regulated in inflammatory bowel disease compared with healthy controls [24]. In murine colitis models, PD-L1-Fc treatment decreased the disease activity and the Th17 cell frequency, together with an increased CD4^+^ T cell-mediated IL-10 production [25]. Taken together, PD-1 function may contribute to the disrupted T cell functions in autoimmune diseases such as CD.

Vitamin D influences the PD-1 pathway by increasing the PD-L1 expression and decreasing the expression of the co-stimulatory receptors CD80 and CD86 following lipopolysaccharides (LPS) stimulation in monocyte-derived dendritic cells (DCs) from healthy controls [26]. In mice, this effect has also been observed in bone marrow-derived DCs [27].

We hypothesized that oral vitamin D treatment modulates PD-1 signalling in CD and aimed to investigate the PD-1 expression in T cells from CD patients who had received oral vitamin D or placebo.

## RESULTS

### Effects of vitamin D treatment in CD patients

**Figure 1:**
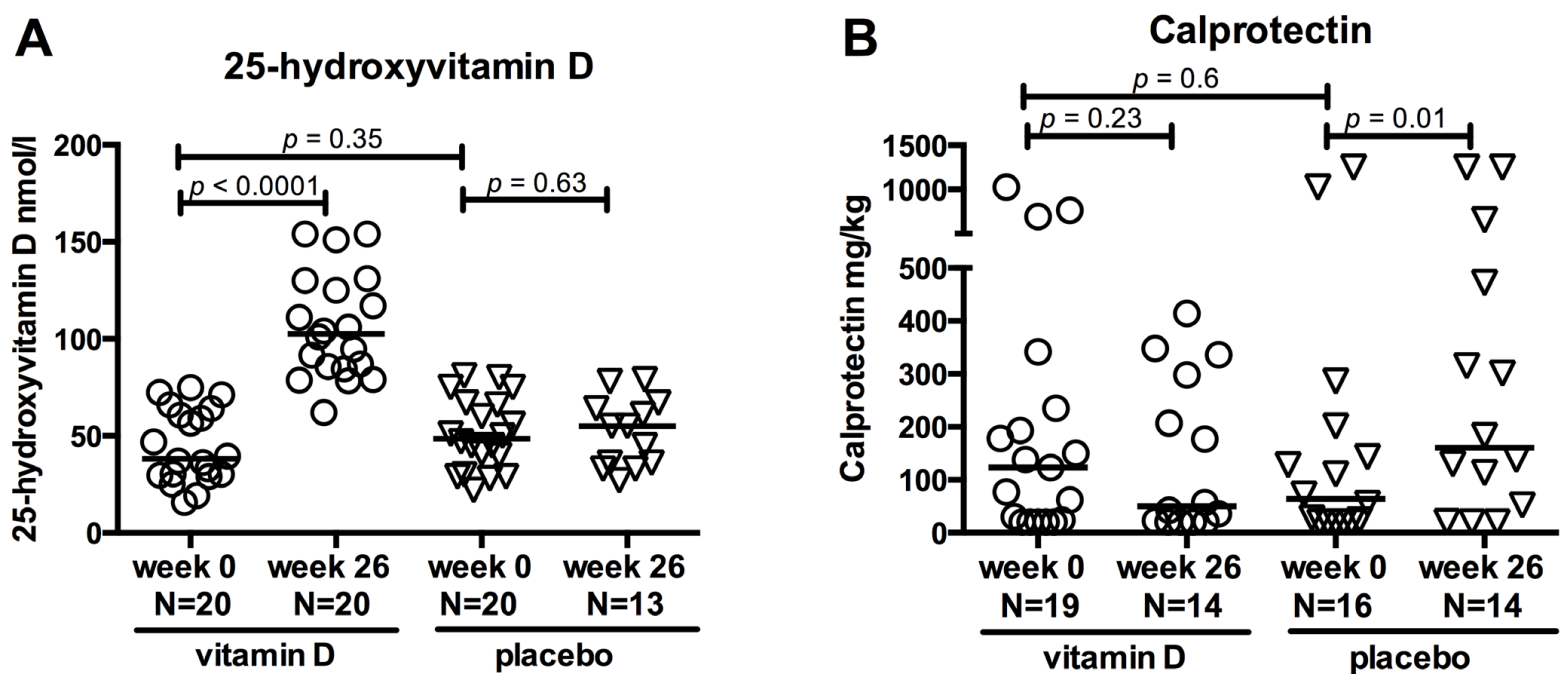
Effects of vitamin D treatment in CD patients Clinical parameters are shown at baseline (0 weeks) and after 26 weeks of treatment with vitamin D or placebo. Circles represent vitamin D treated CD patients and triangles placebo treated CD patients. Solid lines represent medians. **A**. 25-hydroxyvitamin D levels increased in the vitamin D group but not in the placebo group. The vitamin D treated patients were selected on the basis of an increase in 25-hydroxyvitamin D within 26 weeks of treatment. **B**. Faecal calprotectin levels at baseline and after 26 weeks of treatment. Calprotectin levels were unchanged in the vitamin D group but increased in the placebo group. Range from 20 to 1250 mg/kg. N; number.

Baseline patient characteristics of the 40 CD patients included in this study are presented in Table 1. At baseline, patients randomised to vitamin D and placebo did not differ, except from increased use of azathioprine in the vitamin D treated group (*p* = 0.003). The baseline plasma 25-vitD levels were comparable between the two groups (vitamin D group 38 nmol/l (16 - 75), placebo group 49 nmol/l (22 - 81), *p* = 0.35). Baseline 25-vitD levels did not correlate with faecal calprotectin, C-reactive protein (CRP), Harvey-Bradshaw Index (HBI), or Crohn's Disease Activity Index (CDAI) scores (data not shown). In the vitamin D group, 25-vitD levels increased 3-fold from baseline to 26 weeks of treatment (*p* < 0.0001) (Figure 1A) in contrast to no effect on the 25-vitD levels in the placebo group.

**Table 1:** Baseline patient characteristics

	Vitamin D	Placebo	*p* value
*N*	20	20	NA
Male, *n*	11	10	0.75
Age (median, range)	33 (21 – 62)	36 (19 – 66)	0.42
Winter inclusion*, *n*	17	19	0.29
Smokers, *n*	6	8	0.51
Azathioprine users, *n*	12	3	0.003
Biologicals prior to inclusion, *n*	3	3	1.00

Crohn's disease patient characteristics at baseline. The two groups did not differ except for the use of azathioprine where more patients in the vitamin D group used azathioprine compared with the placebo group.

*Winter inclusion; 1^st^ of October to 31^th^ of March, summer inclusion; 1^st^ of April to 30^th^ of September.

Among patients randomised to vitamin D, the CDAI and HBI scores, the biochemical inflammation marker CRP and the mucosal marker faecal calprotectin remained unchanged during the treatment period. Among patients randomised to placebo, faecal calprotectin increased from 64 mg/kg (20 - 1250) to 161 mg/kg (20 - 1250) (*p* = 0.01) after 26 weeks of treatment (Figure 1B). CDAI, too, increased slightly during placebo treatment from 32 (0 - 140) to 42 (12 - 140)(*p* = 0.05). Six out of the 20 placebo patients experienced clinical relapse during one year of follow up while none of the vitamin D treated patients relapsed (*p* = 0.008). Four out of six placebo patients relapsed before week 26 and were excluded from the week 26 analyses. Two out of six placebo patients relapsed later than week 26, and these patients were included in week 26 analyses. Therefore all included patient samples were obtained during remission.

### Vitamin D treatment induces PD-1 expression in activated T cells and changes their activation pattern

**Figure 2:**
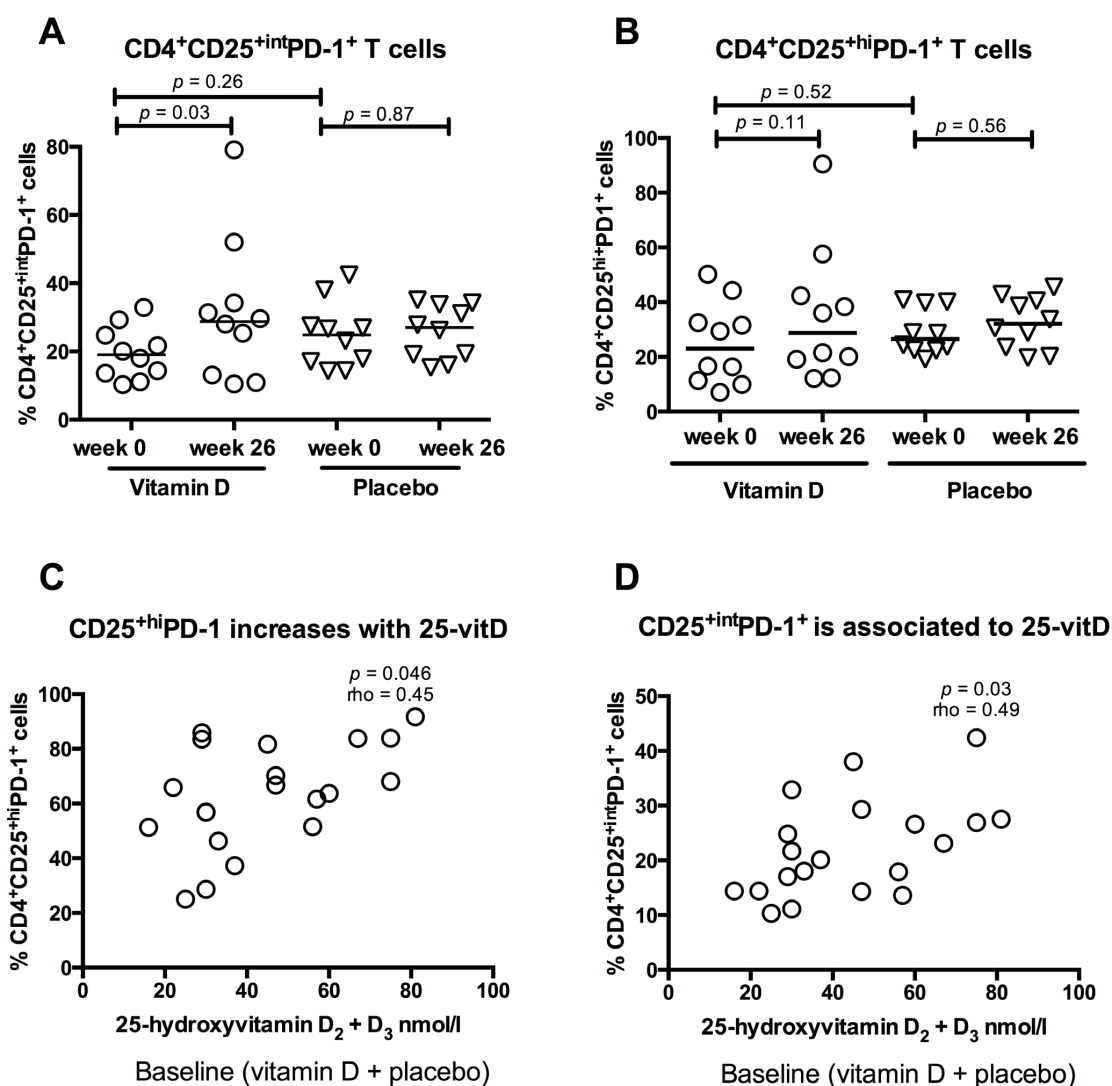
Vitamin D treatment induces PD-1 expression in active T cells Solid lines represent medians, circles represent vitamin D treated CD patients (*N* = 10) and triangles placebo treated CD patients (N = 10). The CD4^+^CD25^+^ population was divided into CD4^+^CD25^+hi^ and CD4^+^CD25^+int^. **A**. Vitamin D treatment increased PD-1 expression in CD4^+^CD25^+int^ T cells compared to placebo treatment. **B**. PD-1 expression in CD4^+^CD25^+high^ cells was not affected by treatment. **C.** 25-hydroxyvitamin D levels are associated with the percentage of CD4^+^CD25^+hi^PD-1^+^ cells at baseline (placebo and vitamin D group, *N* = 20). **D**. 25-hydroxyvitamin D levels are associated with the percentage of CD4^+^CD25^+int^PD-1^+^ at baseline (placebo and vitamin D group, *N* = 20).

Vitamin D treatment did not affect the PD-1 expression in unstimulated or T cell receptor- (TCR) stimulated CD4^+^ and CD8^+^ T cells in general (data not shown). We subsequently focussed our analysis to the subset of activated CD4^+^ T cells expressing the activation marker CD25, which comprised 95% of the total population of CD4^+^ T cells. Because CD25 is highly expressed by regulatory T cells, the CD25 expressing CD4^+^ T cells were further subdivided into regulatory T cell-enriched CD4^+^CD25^+high^ (CD4^+^CD25^+hi^) and activated CD4^+^CD25^+intermediate^ (CD4^+^CD25^+int^) (Figure S1). Vitamin D treatment increased the PD-1 expression upon TCR stimulation, in CD4^+^CD25^+int^ T cells from baseline 19% (range 10 - 39%) to 29% (11 - 79%)(*p* = 0.03), while the expression remained unchanged in patients who received placebo (Figure 2A). PD-1 increase in CD4+CD25+int T cells was not associated to the use of azathioprine (data not shown). The percentage of CD4^+^CD25^+int^ T cells of live cells was not affected by oral vitamin D treatment (data not shown). Neither vitamin D nor placebo treatment influenced the CD4^+^CD25^+hi^ T cells’ PD-1 expression following TCR stimulation (Figure 2B).

To further examine the influence of vitamin D on the PD-1 expression in CD4^+^CD25^+int^ and CD4^+^CD25^+hi^ T cells, levels of CD4^+^CD25^+int^PD-1^+^ and CD4^+^CD25^+hi^PD-1^+^ subsets were correlated to 25-vitD levels at baseline. 25-vitD plasma levels were associated both with the percentage of CD4^+^CD25^+hi^PD-1^+^ cells (p < 0.05, Spearman's rho = 0.45)(Figure 2C) and CD4^+^CD25^+int^PD-1^+^ cells (*p* = 0.03, Spearman's rho 0.49)(Figure 2D).

**Figure 3:**
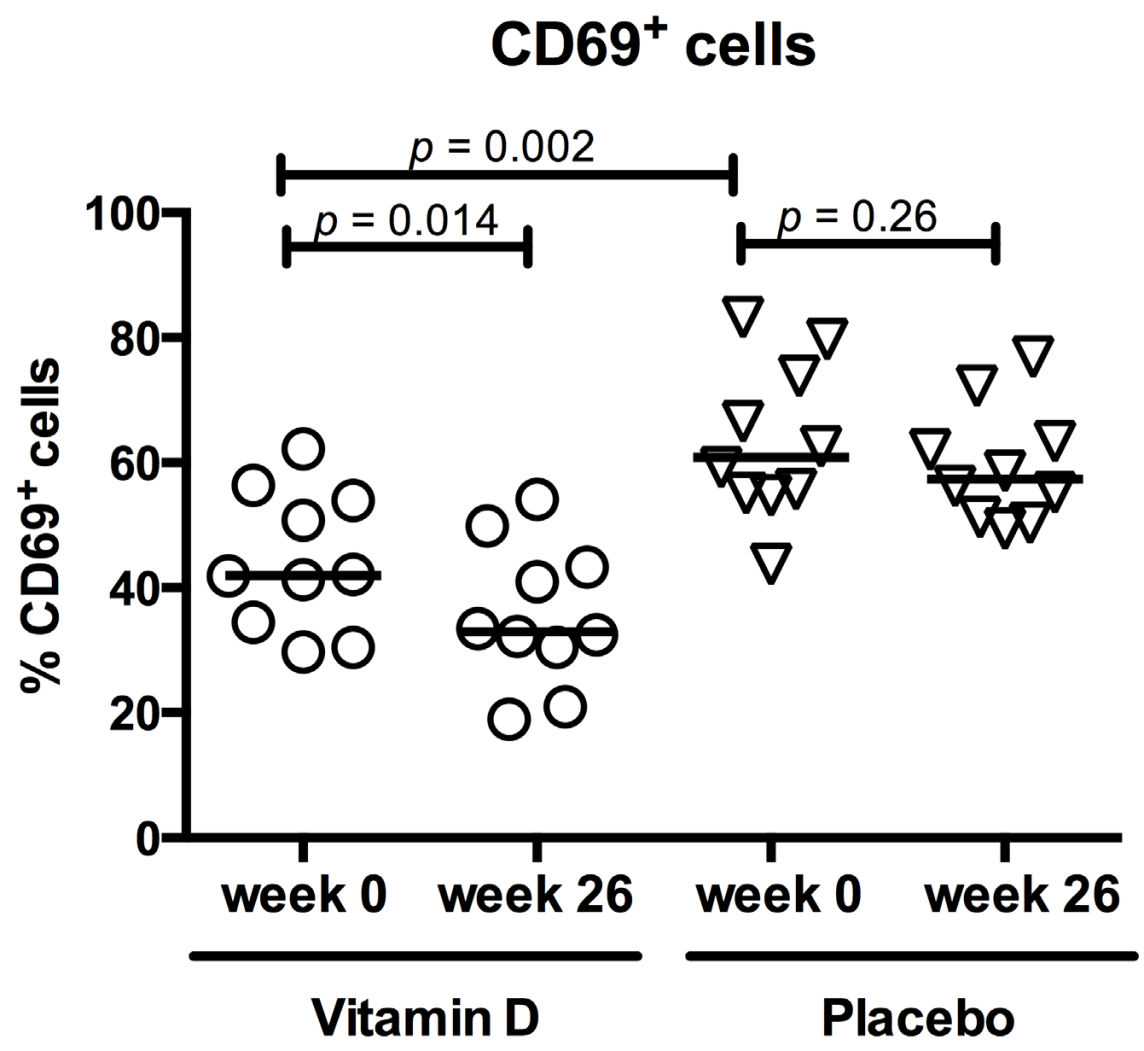
Vitamin D treatment reduced CD69 expression in active T cells Circles represent vitamin D treated CD patients, triangles placebo treated CD patients and solid lines medians. 26 weeks of vitamin D treatment (*N* = 10) reduced the T cells’ ability to up-regulate CD69 upon TCR activation. This was not observed in the placebo group (*N* = 10). At baseline, CD69 expression was higher in the placebo group than the vitamin D group.

The activation pattern of the T cells was changed by vitamin D treatment. In the vitamin D group, CD69 up-regulation upon TCR stimulation was reduced from baseline 42% (31 - 62%) to 33% (19 - 54%)(*p* = 0.01). This was not observed in the placebo group (Figure 3). The baseline CD69 expression was higher in the placebo group compared with the vitamin D group. CD69 expression was not associated with use of azathioprine (data not shown).

### Soluble PD-1 is unaffected by vitamin D treatment

We investigated whether vitamin D treatment affected sPD-1 concentrations in plasma from vitamin D and placebo treated CD patients in remission and from healthy controls (HC) treated with high dose vitamin D for 15 days. Soluble PD-1 was detectable in both CD patients and HC, but plasma levels were clinically unaffected by vitamin D treatment in both CD patients (from 0.092 ng/ml (0.269 - 0.05) to 0.085 ng/ml (0.209 - 0.05), *p* = 0.09) and HC (from 0.072 ng/ml (0.173 - 0.05) to 0.062 ng/ml (0.108 - 0.05), *p* = 0.5) and likewise in placebo treated CD patients (from 0.05 ng/ml (0.155 - 0.05) to 0.05 ng/ml (0.05), *p* = 0.03) (data not shown). Soluble PD-1 levels were not associated with inflammation markers and the disease scores in the group of CD patients at baseline (CDAI, HBI, CRP, and faecal calprotectin)(data not shown).

### 1.25(OH)2D activates T cells and decreases PD-1 expression in cell cultures

**Figure 4:**
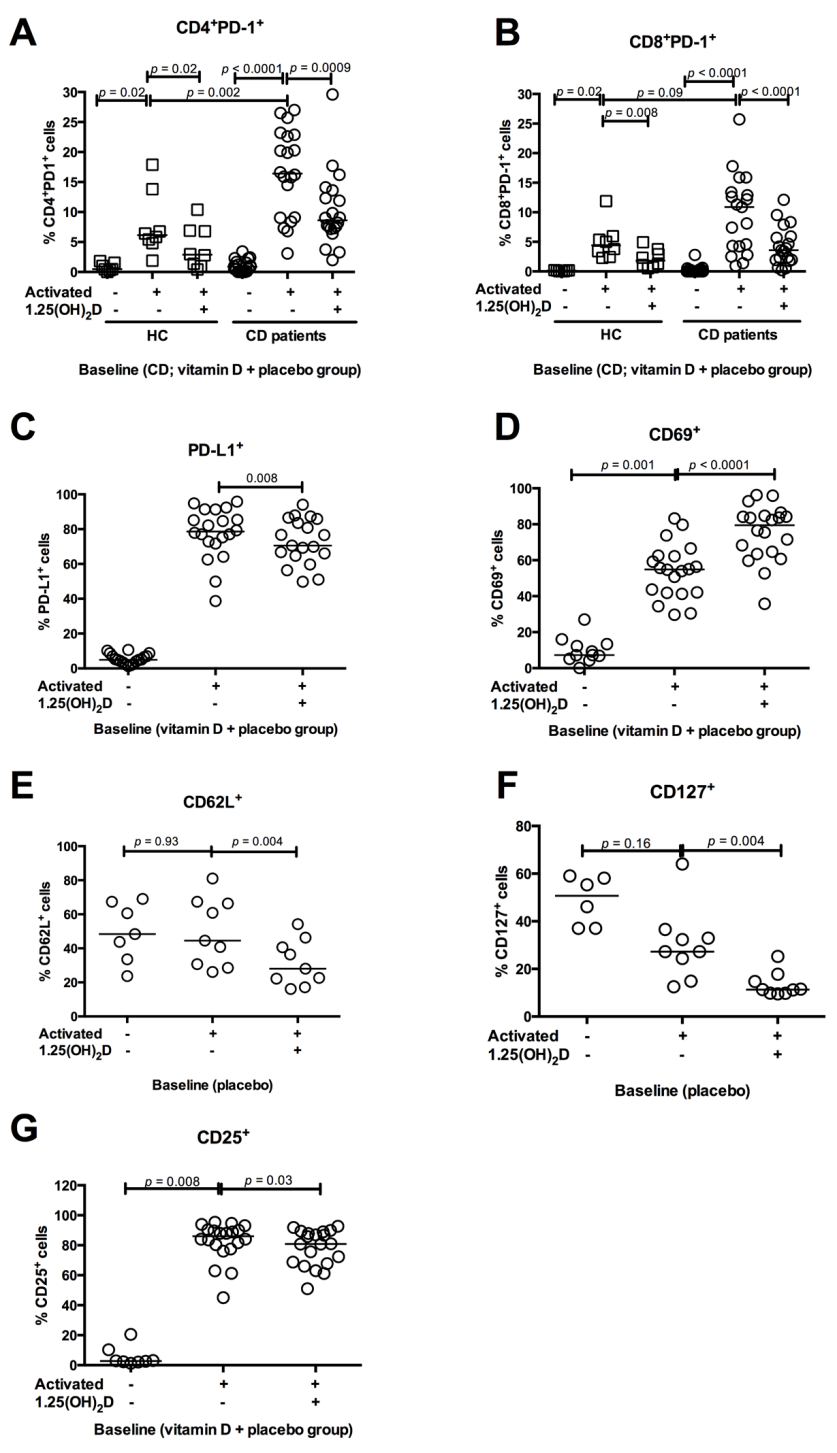
In vitro 1.25(OH)2D decreases PD-1 and activates T cells The influence of *in vitro* 1.25(OH)_2_D stimulation on T cell activation markers and PD-1 from CD patients and HC. Solid lines represent median, squares represent HC and circles CD patients. All figures show baseline data why the CD vitamin D and placebo group are grouped together. **A**. In CD patients, TCR stimulation increased the PD-1 expression in CD4^+^ T cells more than in HC. *In vitro* 1.25(OH)_2_D stimulation decreases the CD4^+^ T cells ability to express PD-1 in both HC (N = 8) and CD patients (*N* = 20). **B**. TCR stimulation increases PD-1 in CD8^+^ T cells similar in HC and CD. *In vitro* 1.25(OH)_2_D stimulation decreases the CD8^+^ T cells´ ability to express PD-1 in both HC (*N* = 8) and CD patients (*N* = 20). **C**. TCR stimulation increases PD-L1 expression in PBMCs from CD patients, while addition of 1.25(OH)_2_D decreases the ability to express PD-L1(*N* = 20). **D**. TCR stimulation increases the CD69 expression, which is further increased by i*n vitro* 1.25(OH)_2_D stimulation in PBMCs (*N* = 20). **E**. CD62L expression was unaffected by TCR stimulation while 1.25(OH)_2_D stimulation decreases the ability to express CD62L in PBMCs from CD patients (*N* = 10). **F**. TCR stimulation did not affect the CD127 expression in PBMCs from CD patients but *in vitro* 1.25(OH)_2_D stimulation decreases the CD127 expression (*N* = 10). **G**. CD25 expression increases when PBMCs are stimulated with TCR. However, 1.25(OH)_2_D stimulation decreased the CD25 expression (*N* = 20). Activated: stimulation with anti-CD3 and anti-CD28, vitamin D; 1.25(OH)_2_D stimulation, HC; healthy controls, vitamin D CD; vitamin D treated Crohn's Disease patients, placebo CD; placebo treated Crohn's Disease patients, PD-1; programmed death 1.

We tested how *in vitro* 1.25(OH)_2_D stimulation affected the PD-1 expression on T cells at baseline. The vitamin D and placebo treated groups were examined together in one group (CD patients). Three days of TCR-mediated stimulation resulted in an up-regulated PD-1 expression on CD4+ and CD8+ T cells in both CD patients and HC. The PD-1 expression in CD4+ T cells increased more in CD patients (from 1% (0 - 3%) to 16% (3 - 27%)) than in HC (from 0% (0 - 2%) to 6% (2 - 18%))(*p* = 0.002) (Figure 4A). In contrast, the CD8^+^PD-1^+^ increase was similar in CD patients and HC (Figure 4B) upon TCR stimulation. Addition of 1.25(OH)_2_D inhibited the PD-1 up-regulation in both CD4^+^ T cells (from 16% (3 - 27%) to 9% (2 - 30%))(*p* = 0.0009) and CD8^+^ T cells (from 11% (1 - 26%) to 4% (0 - 12%))(*p* < 0.0001)(Figure 4A and 4B). This pattern was unaffected after 26 weeks of treatment with vitamin D or placebo (data not shown).

We observed a universal up-regulation of PD-L1 on all peripheral blood mononuclear cells (PBMCs) after TCR stimulation. Addition of 1.25(OH)_2_D reduced the PBMCs ability to up-regulate PD-L1 from 79% (39 - 96%) to 71% (50 - 94%)(*p* = 0.008 Figure 4C). We examined if 1.25(OH)_2_D stimulation affected the T cell activation pattern *in vitro*. Addition of 1.25(OH)_2_D to TCR-stimulated cells increased the T cells expression of CD69 from 55% (31 - 83%) to 80% (36 - 96%)(*p* < 0.0001, Figure 4D), and reduced their expressions of CD62L from 45% (26 - 81%) to 28% (16- 54%)(p = 0.004, Figure 4E) and CD127 from 27% (13- 64%) to 11% (10 - 25%) (*p* = 0.004, Figure 4F). This, together indicates modulation towards a more activated T cell phenotype. However, *in vitro* 1.25(OH)_2_D stimulation resulted in a slightly reduced ability to up-regulate CD25 upon TCR activation from 86% (45 - 95%) to 81% (51 - 93%)(*p* = 0.03, Figure 4G).

The PD-L2 expression was very low (below 1% positive cells) and was unaffected by TCR and 1.25(OH)_2_D stimulation (data not shown). Supernatants from the cell culture (+/− antiCD3/CD28 and +/− 1.25(OH)_2_D) were examined for sPD-1 levels but these were unaffected by 1.25(OH)_2_D stimulation (data not shown).

## DISCUSSION

PD-1 is a novel mediator of immune tolerance. The main finding in this study was an increased PD-1 up-regulation in CD4^+^CD25^+int^ T cells from CD patients who received a daily supplement of vitamin D, compared with CD patients treated with placebo for 26 weeks. Our data indicate that oral vitamin D modulates PD-1 signalling in CD4^+^CD25^+int^ T cells from CD patients.

Vitamin D treatment increased the PD-1 expression in activated CD4^+^CD25^+int^ T cells but not in the Treg-enriched CD4^+^CD25^+hi^ fraction. We have previously shown that vitamin D treatment did not induce CD4^+^CD25^+^FoxP3^+^CD127^−^ Treg in a similar patient cohort [28]. In healthy subjects, it has been demonstrated that vitamin D increases the amount of Tregs [29]. We did observe an association between 25-vitD levels and the amount of CD4^+^CD25^+high^PD-1^+^ T cells at baseline, but this association was not sustained by oral vitamin D or placebo treatment. The TCR- stimulation induced PD1-uregulation in CD4^+^ T cells was more pronounced in CD than in HC, which is line with previous findings in patients with other chronic inflammatory diseases than CD [24, 30].

Upon TCR stimulation, oral vitamin D treatment to CD patients increased the up-regulation of PD-1 in CD4^+^CD25^+int^ T cells and inhibited the T cell activation pattern, indicated by a reduced CD69 up-regulation. This finding is in accordance with a study in healthy volunteers, where two months of high dose, daily vitamin D treatment also inhibited the CD4^+^ T cell's activation evaluated by a decreased adenosintriphosphat (ATP) release [31]. Our results suggest that oral vitamin D treatment to CD patients may mediate reduced T cell activation by increasing the PD-1 expression in CD4^+^CD25^+int^ T cells.

We did not observe an association between the soluble PD-1 concentration and disease activity scores as observed in RA patients with active disease [19]. In the present study, all patients were in remission and were thus not comparable with the studied RA patients. The observed increased PD-1 expression in CD4^+^CD25^+int^ T cells was not associated with an altered shedding of the PD-1 receptor. Our findings support data from the clinical study [6] where vitamin D inhibited inflammation and reduced the risk of relapse. We, and others have previously demonstrated an association between low 25-vitD levels and increased disease activity scores [32, 33] and faecal calprotectin levels [34]. These data support that vitamin D has immunoregulatory effects.

We also examined the effects of *in vitro* 1.25(OH)_2_D stimulation. Here, 1.25(OH)_2_D increased the activation of TCR-stimulated T cells, demonstrated by a decrease in CD127 and CD62L expressions and an increase in CD69 expression. Interestingly, PD-1 expression was strongly reduced by *in vitro* 1.25(OH)_2_D stimulation. This suggests that T cells respond differently to *in vitro* 1.25(OH)_2_D stimulation than to 26 week of *in vivo* vitamin D treatment. This may reflect that the *in vitro* conditions do not represent the *in vivo* setting. Alternatively, vitamin D could exert a dualistic regulation of T cells, initially activating the T cells and over time be switching to an inhibition of the T cell activation. This is not possible to decipher in the present study. Evidence to support the latter hypothesis remains ambiguous. Von Essen et al. showed that the phospholipase C-dependent activation pathway of T cells depended on vitamin D activation [35]. Others have suggested that *in vitro* 1.25(OH)_2_D stimulation decreases CD4^+^CD25^−^ T cells proliferation [30].

In the present study we used high CD25 expression to enrich for Tregs in our analysis. However, the discrimination of Tregs from activated T cells, solely by CD25 can be difficult [36, 37]. Just like other Treg markers such as FoxP3^+^ and CD127^−^, CD4^+^CD25^+high^ expression is not exclusive to Tregs, although the proportion of Tregs is considerably higher among the CD4^+^CD25^+hi^ subset, than in the CD4^+^CD25^+int^ subset.

One important limitation in the present study is that our findings could be confounded by the increased use of azathioprine in the vitamin D treated group. In the original clinical trial, more patients received azathioprine in the vitamin D-treated group than in the placebo-treated group, but the difference was not statistically significant. Adjustment for use of azathioprine only changed the risk estimates slightly [6]. In the present study, we observed no associations between azathioprine use and cell surface markers, pointing to vitamin D treatment as the regulator of the expression of the investigated cell surface markers.

In conclusion, oral vitamin D treatment in CD patients increases the PD-1 expression in CD4^+^CD25^+int^ T cells and decreases the CD69 expression. This may reflect that vitamin D influences immune tolerance through the PD-1 mediated modulation of T cell responses in Crohn's disease.

## MATERIALS AND METHODS

### Participants

A total of 108 CD patients were included in a randomised placebo-controlled clinical trial [6]. Each patient was treated daily for one year with 30 μg vitamin D3 and 1200 mg calcium or placebo and calcium. In the present study, a subgroup was selected from the clinical trial. Twenty vitamin D3 treated patients were included, on the basis of a baseline 25-vitD serum level below 85 nmol/l combined with an increase in 25-vitD from baseline to week 26. Another 20 patients who had been randomised to placebo and who had baseline 25-vitD levels below 85 nmol/L were included as a control group. Other patient characteristics were independent of the selection. Plasma samples from week 26 were available from all vitamin D-treated patients, but only from 11 of the placebo patients, due to drop-out (*N* = 3), exclusion because of relapse within 26 weeks (*N* = 4), and unavailable patient samples (*N* = 2). Plasma samples were isolated at week 0 and 26 and stored at -140 °C. Peripheral blood mononuclear cells (PBMC) were examined from 10 vitamin D and 10 placebo treated CD patients from the selected groups. PBMCs were isolated by density gradient separation (Ficoll-Paque, Amersham Biosciences, Uppsala, Sweden) at baseline and week 26, and stored at -140°C.

In both groups, the following inflammation markers and clinical scores were measured at weeks 0 and 26: faecal calprotectin, CRP, CDAI [38, 39] and HBI [40].

Eight HC were included for the examination of surface markers on PBMCs. PBMCs were isolated and stored as described above. An additional ten healthy volunteers were included in order to measure soluble PD-1 in plasma. All HC were treated with a high dose of vitamin D (bolus of 5 mg vitamin D3 followed by 0.5 mg vitamin D3 for 14 days). Plasma was isolated at day 0 and 15 and stored at -140°C.

### Culture of PBMCs

PBMCs were thawed and diluted in RPMI 1640 and washed twice. Cells (1 × 10^6^/ml) were cultured in RPMI-10 medium (1 × 10^6^ cells/well)(RPMI 1640, penicillin and streptomycin, 10% human heat-inactivated AB serum, and 1.5 ml HEPES) and stimulated with or without 0.1 μg/ml immobilised anti-CD3 (Orthoclone OKT3; a kind gift from Cilag AG International, Schaffhausen, Switzerland) and with or without 10^−8^ M 1.25 dihydroxyvitamin D_3_ (Sigma-Aldrich, St Louis, MO, USA). One hour later 0.05 μg/ml soluble anti-CD28 (cat. no. 55BD Biosciences, San Diego, CA, USA) was added. PBMCs were cultured in 24-well plates (TPP, Trasadingen, Switzerland) for three days. Day three, the PBMCs were harvested, washed and suspended in PBS (5 × 10^6^/ml). 100 μl of the PBMC-suspension was stained with the surface markers: CD4 (cat. no. 555346, BD Pharmingen), CD8 (cat. no. 555369, BD Pharmingen), CD14 (cat. no. 345784, BD Bioscience), CD19 (cat. no. 557791, BD Pharmingen), CD25 (cat. no. 557741, BD Pharmingen), CD69 (cat. no. 310910, BioLegend), PD-1 (cat. no. 557946, BD Pharmingen), PD-L1 (cat. no. 329718, BioLegend), PD-L2 (cat. no. 557926, BD Pharmingen), CD62L(cat. no. 304816, BD Pharmingen), CD127(cat. no. 557938, BD Pharmingen) and Via Probe (cat. no. 555815, BD Bioscience), and incubated 30 minutes in the dark at 4°C. The cells were subsequently washed and resuspended in 250 μl fixating buffer (PBS, 1% formaldehyde). Cells were analysed by flow cytometry within 24 hours. The CD4^+^CD25^+^ population was divided into CD4^+^CD25^+ high^ (CD4^+^CD25^+hi^, the 5% of cells with the highest CD25 expression) and CD4^+^CD25^+ intermediate^ (CD4^+^CD25^+int^, the remaining 95% of CD4^+^CD25^+^ T cells). Then PD-1 expression was analysed in each cell subset (Figure S1). Only live, single cells were included and gates were based on isotype or FMO controls.

### Enzyme-linked immunosorbent assay (ELISA)

A sandwich ELISA was used to quantify plasma and supernatant levels of sPD-1. sPD-1 levels were analysed according to the manufacturer's instructions using DuoSet Human PD-1 (R&D systems, Minneapolis, MN). Plasma samples were diluted 1:2 in Reagent dilution (phosphate-buffered saline (PBS), 40 μg/ml bovine antigen (Jackson Immunoresearch, Suffolk, UK) and 20 μg/ml goat antigen (Jackson) to ensure pre-aggregation of heterophilic antibodies in the samples. Supernatant samples were not diluted. The minimum detection limit was 0.05 ng/ml, and values below the detection limit were assigned the value of the detection limit. Samples were analysed in duplicate and the average optical density (OD) values were used to determine the concentrations. The sPD-1 ELISA has previously been validated [19].

### Faecal calprotectin

Calprotectin levels (mg/kg) were quantified in stool samples with the ELISA kit Phical Test/Calprotectin (NovaTec Immundiagnostica GmbH, Dietzenbach, Germany). The range was from 20 to 1250 mg/kg. The levels 20 represented levels ≤ 20 mg/kg and the given levels at 1250 represented levels ≥ 1250 mg/kg (outliers were truncated).

### Statistics

Comparison of baseline characteristics between the vitamin D and placebo treated group were analysed with Chi square test or Wilcoxon rank sum test. We used nonparametric Wilcoxon rank sum test to compare unpaired data and Wilcoxon signed rank test with paired data. Results are presented as median with range. An association between two variables was estimated using Spearman's rank correlation coefficient (Spearman's rho). A p-value below 0.05 was considered statistically significant. All the statistical analyses and graphs were completed using GraphPad Prism version 6 (GraphPad Software Inc., La Jolla, CA, USA).

### Ethics

The clinical study (which provided patient samples to the present study) and additional studies based on the collected patient samples were approved by the National Committee on Health Research Ethics (j. no. 2004/0149) and the Danish Medicines Agency (EudraCT no. 2005/001216/50) and was registered at ClinicalTrial.gov (NCT 0013 2184). In the randomized clinical trial, the patients signed written informed consent and approved that blood samples were stored for further studies on the effects of vitamin D in CD. The clinical trial conformed to the Helsinki Declaration and to Danish legislation. Patients gave informed consent to participate in the study [6]. The 10 vitamin D-treated healthy controls are controls in an on-going clinical study, which has been approved by the regional Committee of Ethics (j.no. 51462) and the Danish Medicines Agency (EudraCT no. 2013-000971-34). Healthy controls gave informed consent to participate in the study and signed and approved that blood samples were stored for further studies on the effects of vitamin D in healthy controls.

## SUPPLEMENTARY MATERIALS FIGURES



## Abbreviations

CD: Crohn's Disease
HC: healthy controls
1.25(OH)2D: 1.25-dihydroxyvitamin D3
25-vitD: 25-hydroxyvitamin D
PD-1: programmed death 1
PD-L1: programmed death ligand 1
PD-L2: programmed death ligand 2
PBMCs: peripheral blood mononuclear cells
IL: interleukin
TNFγ: tumour necrosis factor alpha
IFN-γ: interferon gamma
CDAI: Crohn's disease activity index
HBI: Harvey-Bradshaw index
CRP: C-reactive protein
TCR: T cell receptor
CD4+CD25+hi: CD4+CD25+high
CD4+CD25+int: CD4+CD25+intermediate
ATP: adenosintriphosphat
LPS: lipopolysaccharides
Treg: regulatory T cells
Th17 cells: T helper 17 cells.
