# Supplementary material for: Vitamin D increases programmed death receptor-1 expression in Crohn’s disease
Source: Oncotarget. 2017 Feb 18;8(15):24177–86. doi: 10.18632/oncotarget.15489 (PMC5421837; doi:10.18632/oncotarget.15489)
Supplement: Supplementary file 1 [file oncotarget-08-24177-s001.pdf]

Vitamin D increases programmed death receptor-1 expression in Crohn's disease

Supplementary Material

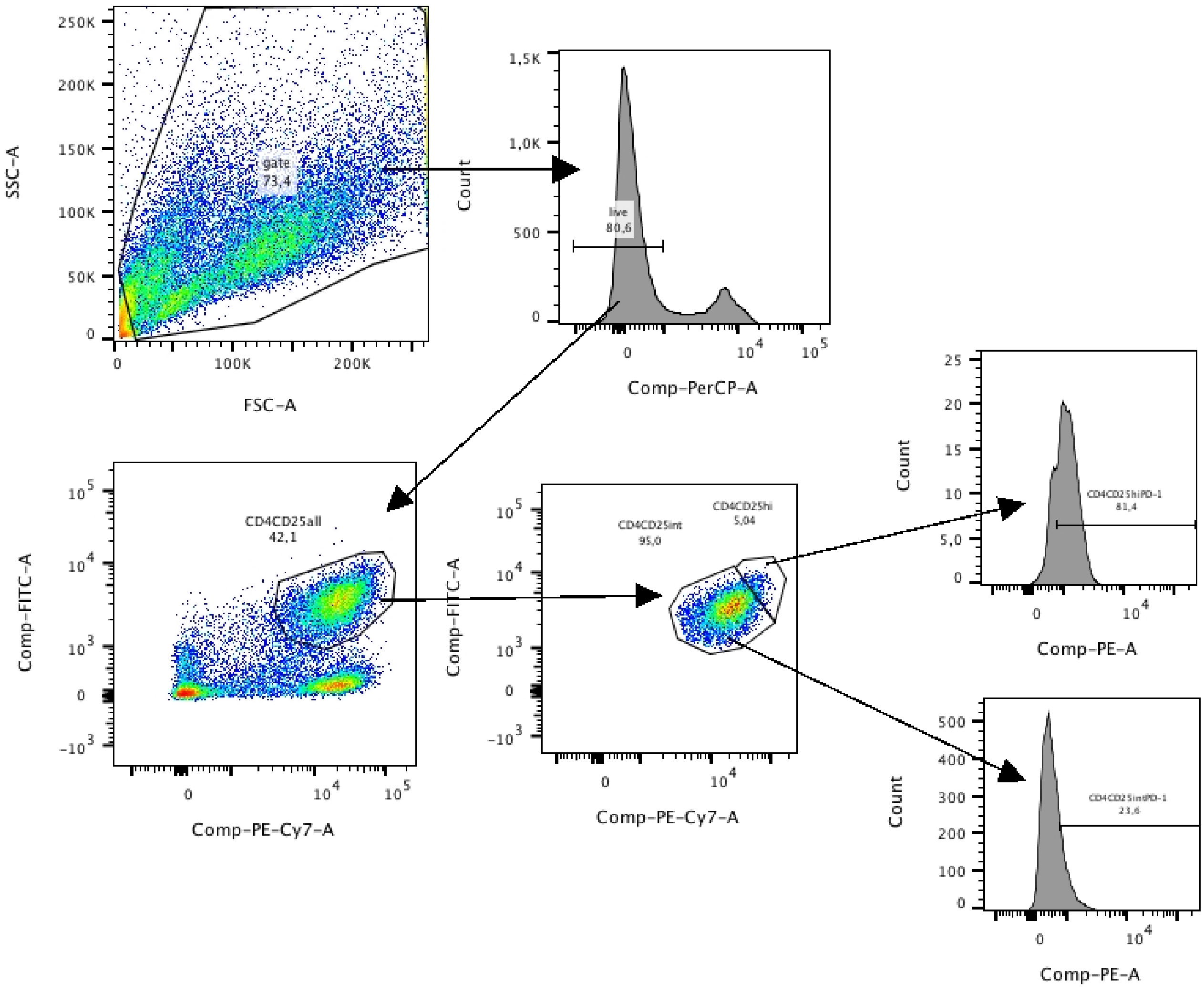

Figure S1: CD25 gating strategy. Gating strategy of a representable patient for dividing CD4^{+}CD25^{+} T cells into CD4^{+}CD25^{+hi} and CD4^{+}CD25^{+int}. Hereafter PD-1 expression was examined in the two subsets. The PD-1 gate was set out from an isotype control.
